# Interleukin-7 Biology and Its Effects on Immune Cells: Mediator of Generation, Differentiation, Survival, and Homeostasis

**DOI:** 10.3389/fimmu.2021.747324

**Published:** 2021-12-02

**Authors:** Deng Chen, Ting-Xuan Tang, Hai Deng, Xiang-Ping Yang, Zhao-Hui Tang

**Affiliations:** ^1^ Division of Trauma and Surgical Critical Care, Department of Surgery, Tongji Hospital, Tongji Medical College, Huazhong University of Science and Technology, Wuhan, China; ^2^ Class 1901, School of Medicine, Wuhan University of Science and Technology, Wuhan, China; ^3^ Department of Immunology, Tongji Medical College, Huazhong University of Science and Technology, Wuhan, China

**Keywords:** IL-7, immune cells, generation, differentiation, survival, homeostasis

## Abstract

Interleukin-7 (IL-7), a molecule known for its growth-promoting effects on progenitors of B cells, remains one of the most extensively studied cytokines. It plays a vital role in health maintenance and disease prevention, and the congenital deficiency of IL-7 signaling leads to profound immunodeficiency. IL-7 contributes to host defense by regulating the development and homeostasis of immune cells, including T lymphocytes, B lymphocytes, and natural killer (NK) cells. Clinical trials of recombinant IL-7 have demonstrated safety and potent immune reconstitution effects. In this article, we discuss IL-7 and its functions in immune cell development, drawing on a substantial body of knowledge regarding the biology of IL-7. We aim to answer some remaining questions about IL-7, providing insights essential for designing new strategies of immune intervention.

## Introduction

Interleukin-7 (IL-7) was discovered in the last century and noted for its growth-promoting effects on progenitors of B cells *in vivo* (1). It was subsequently shown that IL-7 is a 25-kDa soluble globular protein. IL-7 is produced by cells, such as fetal liver cells, stromal cells in the bone marrow (BM), and thymus and other epithelial cells, including keratinocytes and enterocytes (2). IL-7R is a heterodimeric complex consisting of the α-chain (CD127) and the common cytokine receptor γ-chain, shared with the receptors for IL-2, IL-4, IL-7, IL-9, IL-15, and IL-21, and expressed in a variety of cells (3). Thus, IL-7 has multiple biological activities and influences various cell types through binding to its receptor. Deficiencies in IL-7 or IL-7R can lead to severely impaired immune cell development (**Table 1**). In the ensuing decades, the discovery of relevant signaling pathways was accompanied by recognition that IL-7 plays an indispensable role in the development and maintenance of many other immune cells. The vital regulatory functions of IL-7 throughout the entire immune system have become increasingly evident.

**Table 1:** The effects of deficiency of IL-7 and its receptor on development of immune cells.

Cells	Effects	Treatment with IL-7
**Thymus**	Decrease in thymic cell count Thymic involution	Increase in thymic cell count Recovery of thymic function
**T cells**	Inhibition of glucose metabolism Cell atrophy Impairment of T-cell functions Severe impairment of T lymphopoiesis T-cell apoptosis	Restoring T-cell numbers Increasing the diversity of T cells Boosting T-cell function Inhibiting T-cell apoptosis Promoting glucose metabolism Preventing T-cell from atrophy
**B cells**	Block in transition to pro-B cells in the BM Impairment of B differentiation potential Impairment of early B lymphopoiesis B-cell apoptosis	Increase in B-cell numbers Allowing the transition of pro-B cells Promoting B-cell survival Increasing antibody production
**NK cells**	Decrease in CD56^bright^NK cell count Impairment of functional responsiveness Pronounced reduce of NK cell cytotoxicity	Increase in NK cell count Promoting survival of CD56^bright^NK cells Inducing pronounced enhancement of NK cell cytotoxicity
**ILCs**	Impairment of ILC differentiation and generation	Increase in ILC numbers Achieving the entry of lymphocytes into lymph nodes
**Monocytes/macrophages**	Inhibition of monocyte activity Reduce of cytokine secretion	Increasing antigen presentation Augmenting the activity of monocytes Promoting cellular proliferation Increasing cytokine secretion Inducing the recruitment of monocytes
**Dendritic cells**	Decrease in DC count	Continuous generation of functional dendritic cells Creating microenvironments for thymic DCs
**Neutrophils**	Decrease in cell count Recruitment delay of neutrophils	Increase in neutrophil count Accelerating the recruitment of neutrophils
**Eosinophils**	Reduced production of eosinophils Inhibition of eosinophil survival	Increase in **e**osinophil numbers Promoting the survival of **e**osinophils

### IL−7−Mediated Signaling Pathways

IL-7Rα is expressed in early thymocytes, T cells, pre-B cells, BM macrophages, and other immune cells. In these cells, IL−7−mediated signaling initiates downstream signaling pathways through Janus kinase 1 (JAK1), JAK3, and phosphoinositide 3−kinase (PI3K), which further leads to the activation and phosphorylation of signal transducer and activator of transcription 5 (STAT5). Phosphorylation of the IL-7Rα chain is critical for the next stage of signal transduction because it contributes to the recruitment of STAT proteins. Phosphorylation of STAT proteins makes it possible for them to dimerize and translocate to the nucleus, where they act as transcription factors for target genes by binding to specific promoter elements. This results in changes in the expression of B-cell lymphoma 2 (Bcl−2) family members, such as increased expression of the anti−apoptotic molecules Bcl-xl, Mcl-1, and Bcl−2 and decreased expression of the pro−apoptotic molecules Bax, Bim, and Bad.

**Figure 1:**
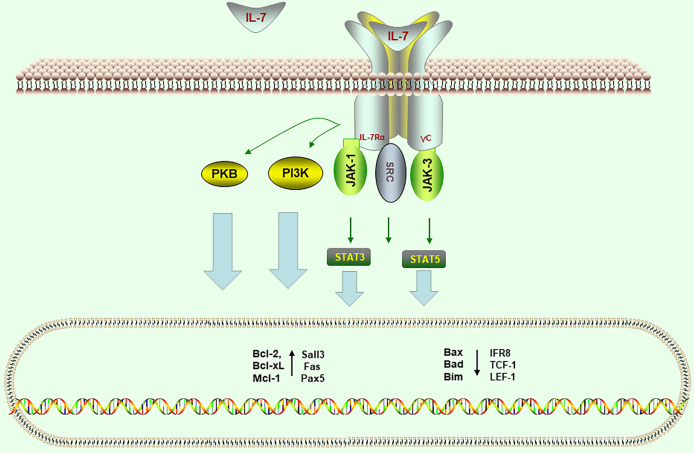
Signal transduction pathways of interleukin-7 receptor (IL-7R). Downstream signaling pathways induced by IL-7 involve Janus kinase 1 (Jak1), Jak3 (through the g-chain), Src kinases, phosphatidylinositol-3 kinase (PI3K), phosphokinase B (PKB), STAT3 (signal transducer and activator of transcription 3), and STAT5. Signal transduction induces changes of gene expression levels in the nucleus, including promoting anti-apoptotic factors (such as Bcl-2, Bcl-xL, and Mcl-1) and inhibition of pro-apoptotic factors (such as Bax, Bad, and Bim).

Src family kinases are also activated by IL-7 binding. These kinases play an important role in developing B cells, but their function in IL-7 signaling has not been fully elucidated. Studies revealed that a potential function of Src kinases is to help activate STAT proteins because they can be phosphorylated by Src kinases independently or in conjunction with JAK proteins (4). One key downstream mediator of PI3K signaling is the serine/threonine kinase Akt (PKB). Akt serves as a central modulator of normal and aberrant B-cell differentiation *via* regulation of variety of pro- and anti-apoptotic factors (5). The molecular structure and signal transduction pathways of IL-7R are shown in **Figure 1**.

**Figure 2:**
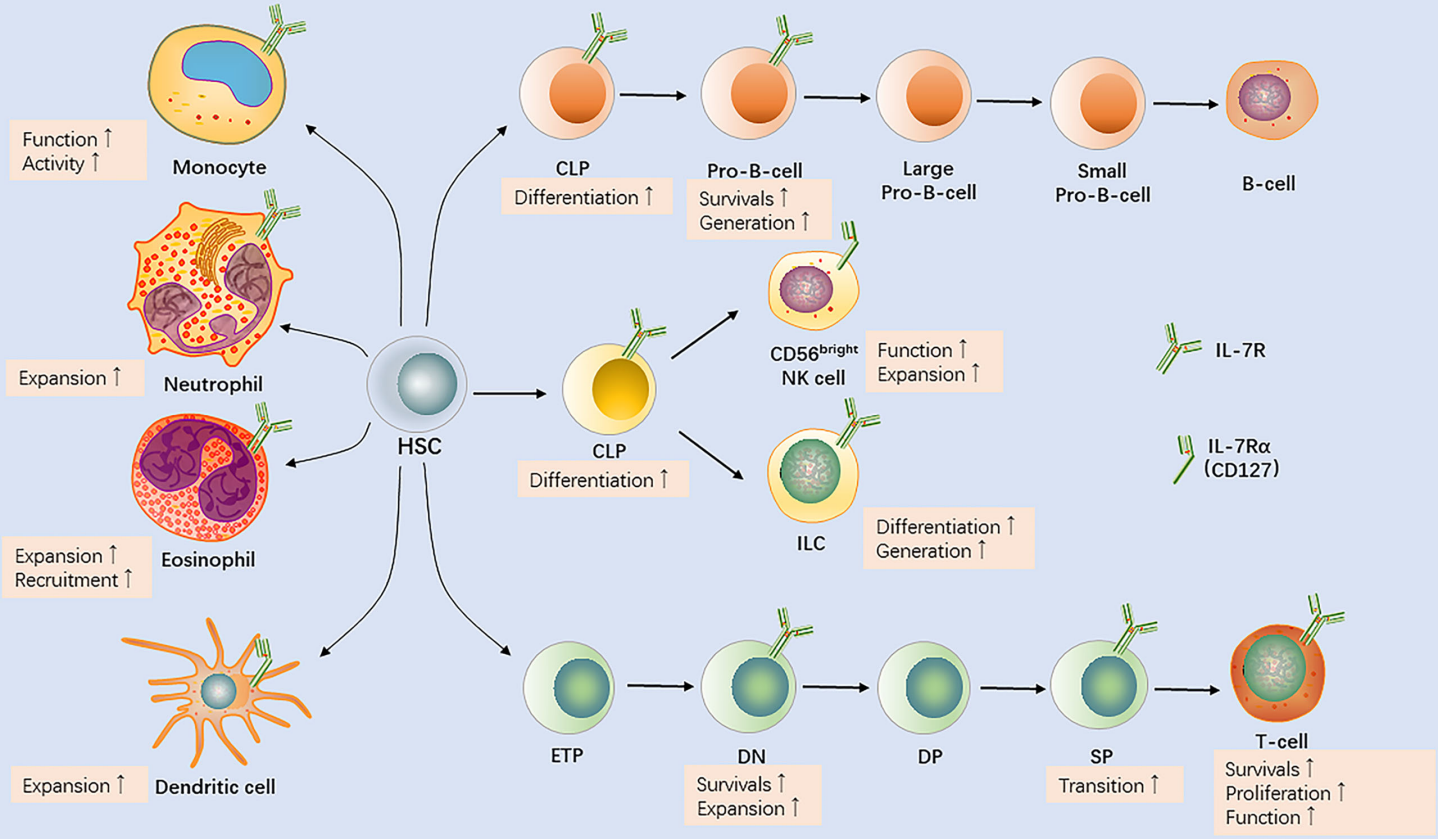
IL-7R expression by immune cells and the effects of interleukin-7 (IL-7) on the development of T cells, B cells, natural killer (NK) cells, innate lymphoid cells (ILCs), monocytes/macrophages, dendritic cells, neutrophils, and eosinophils. HSC, hematopoietic stem cell; CLP, common lymphoid progenitor; ETP, early T-cell lineage progenitor; DN, double−negative; DP, double−positive; SP, single−positive.

Beyond contribution to homeostasis of peripheral T cells, elevated production of IL-7 promotes survival of both naïve and memory T cells (6). IL-7 was suggested to be involved in multiple stages of the development of B-cell progenitor, including its commitment, survival, differentiation, and proliferation (7). Moreover, IL-7 is a non-redundant cytokine with the ability to regulate the recruitment of leukocytes such as neutrophils and monocytes (8, 9). The results of animal experiments and clinical findings suggest that IL-7 is required to maintain and develop immune cells. In this review, we discuss IL-7 and its functions in immune cell development based on the body of knowledge regarding IL-7 biology (**Figure 2**), with the aim of answering the remaining questions, essential for the design of new immune intervention strategies.

## IL-7/IL-7R and T cells

Although IL-7 originally was discovered for a novel molecule acting exclusively on B cells, many critical biological activities of T cells are susceptible to IL-7.

### IL-7/IL-7R and T-Cell Lymphopoiesis

IL-7 signaling is necessary for the development of T cells. Defects in IL-7 or IL-7 receptors in humans lead to severe impairment of T lymphopoiesis (10). IL-7 plays a unique role in the development of murine T cells, demonstrated by the paucity of lymphocytes present in IL-7- and IL-7R-deficient mice and following IL-7 or IL-7R neutralization *in vivo*. In severe combined immunodeficiency (SCID) resulting from mutations in JAK3, T cells were obviously decreased. JAK3 is indispensable for gamma(c)-dependent signaling because it encodes a Janus family tyrosine kinase that couples gamma(c), indicating that defects in IL-7Ralpha signaling caused T^-^B^+^NK^+^ SCID (10).

The biological effects of IL-7 on T-cell lymphopoiesis vary for different lineages during the stages of differentiation. Recent studies have shown that the number of early thymic progenitors (ETPs) in mice with impaired IL-7 signaling was significantly decreased, while the number of ETPs in mice with overexpression of IL-7 was greatly increased (11). The findings indicate that IL-7 can promote the development of ETPs. In addition, IL-7 was indispensable during the γδTCR (T-cell receptor) rearrangement because γδT cells were completely absent from IL-7^-^ mice. However, when the effect of IL-7 is limited, other elements may compensate. For instance, thymic stromal lymphopoietin (TSLP) also signals through the IL-7R subunit, which can substitute for IL-7 in thymopoiesis to stimulate the proliferation of CD4^+^ single-positive thymocytes and peripheral T cells, although this is a suboptimal choice. Beyond playing a critical role in the T lineage progenitor stage of thymopoiesis, IL-7 is also crucial for developing double-negative (DN) thymocytes. IL-7 is an efficient growth factor for DN thymocytes and may serve as an amplification step during thymopoiesis. In early thymocytes, IL-7R signals function nonredundantly by promoting proliferation and survival of CD44^+^25^+^DN thymocytes (so-called DN2 cells) (12, 13). Work by Munitic et al. showed that beyond the DN stage, the forced expression of IL-7Rα could lead to a diminished size of the DN pool. The researchers suggested that this may occur due to the consumption of IL-7, which then contributed to a reduced supply of IL-7 available for DN thymocytes (14). In contrast to the rigorous requirements for IL-7 signaling in double-negative stage 2 (DN2) thymocytes, IL-7 signaling is commonly extinguished by the immature single positive (ISP) stage of thymocyte development. Notably, work by Yu et al. demonstrated that IL-7R signals act as inhibitors of TCF-1, LEF-1, and RORgammaT, all of which are essential for the transition of DP to SP in the thymus (15). Regulation of IL-7R is also significant during the double-positive stage of development because IL-7R is indispensable for transforming signaled double-positive thymocytes into functionally mature CD8^+^ T cells (16).

Evidence suggests that IL-7 is an essential requirement for normal thymopoiesis. This raised the possibility that diminished IL-7 production could result in thymic involution and that IL-7 treatment may promote thymopoiesis in lymphopenic individuals. Many researchers have pursued this appealing hypothesis. Although IL-7 therapy in aged mice could not reverse their thymic involution, some reports showed that IL-7 therapy can gradually accelerate recovery of thymic function (17). The multiple functions of IL-7 in T-cell lymphopoiesis have encouraged researchers to apply IL-7 as a therapy for recovering T-cell numbers (18–21). In addition to the application of IL-7 alone, combining IL-7 with other molecules holds potential and has attracted interest. Mamoru et al. demonstrated that when IL-7 was induced in the presence of IL-12, the diversity of intratumoral CD8+ T cells increased, and IL-12 function was augmented to promote clonality (22). Similarly, immunocomplexes of IL-7 and αIL-7 mAb M25 (IL-7/M25) were described as super-agonists, remarkably augmenting the size of the T-cell pool. Moreover, the immunocomplex effectively shifted the CD4^+^:CD8^+^ T cell ratio in favor of CD8^+^ T cells (23). These studies demonstrate that applying IL-7 to clinical therapeutics effectively boosts T-cell function and restores T-cell numbers to re-establish immune competence (24).

### IL-7/IL-7R and T-Cell Survival

Without external disturbance, the T-cell homeostasis in the peripheral lymphoid compartment is rigorously regulated through turnover, survival, and death. By itself, tonic TCR signaling is not sufficient to keep T cells alive. IL-7 promotes T-cell survival by upregulating the expression level of the Bcl-2 family of molecules, especially Mcl-1 and Bcl-2, which can extensively inhibit the mitochondrial apoptotic pathway. Acting as a critical anti-apoptotic factor, Mcl-1 plays an important role in the survival of single-positive thymocytes, DN thymocytes, naïve T cells, and activated T cells. Moreover, Mcl-1 functions together with Bcl-xL to promote double-positive thymocyte survival (25). However, some reports challenged the conclusion that IL-7 regulates the expression of two anti-apoptotic factors in peripheral T cells because experiments with CD127 conditional deficient mice revealed no distinct effect on the level of Mcl-1 and Bcl-2 expression compared with normal mice for 3 days, indicating that IL-7 signaling was not required to regulate these molecules (26, 27). However, a major limitation is that the half-life of both anti-apoptotic factors could be longer than 72 h.

In addition to dependence on a dynamic balance of pro-apoptotic and anti-apoptotic signals, it should be emphasized that the capacity of IL-7 to maintain steady metabolism—especially glucose metabolism—is also critical for T-cell survival (28). Previous reports have validated that IL-7 promotes glucose metabolism *in vitro* to prevent T-cell atrophy (29). Once stimulated by growth factors, T cells increase their rate of glucose uptake and glycolysis. This function is mediated *via* a signaling mechanism in which STAT5 transcriptional activity promotes Akt activation to regulate glucose uptake and glucose transporter 1 (Glut1) trafficking, essential for IL-7 to prevent T-cell death and maintain homeostasis (30). T cells generally shrink and undergo atrophy when they were transferred into IL-7-deficient hosts (29). Although inhibition of CD127 expression on normal mature T cells did not cause evident changes in total Glut1 levels and glucose uptake, it reduced the rate of glycolysis and induced cell atrophy (26). Taken together, findings demonstrate that IL-7R signaling is essential for promoting T-cell survival through regulating glycolysis. More recently, scientists tried to apply IL-7 to CAR-T cells given the great success chimeric antigen receptor (CAR)-engineered T cells showed in cancer treatment. Surprisingly, they found that expression of IL-7 and CCL19 significantly improved T-cell infiltration and survival of CAR-T cells in mouse tumors, enhancing the anti-tumor activity against solid tumors (31).

## IL-7/IL-7R and B Cells

### IL-7/IL-7R and B-Cell Lymphopoiesis

Hematopoietic stem cells (HSCs) naturally differentiate into B cells. During the process, cells gradually demonstrate B-cell traits but inhibit the traits of other lineages. IL-7 exerts important functions in mouse B cell development, exemplified by the fact that mice with IL-7 deficiency lack both pre-B cells and mature B cells (13, 32). An experiment with IL-7R^-^ mice detected reduced expression levels of Pax5 in BM cells. Pax5 acted as an essential transcription factor in early B lineage cells (33). More importantly, common lymphoid progenitors (CLPs) lose the ability to differentiate into B220^+^CD19^+^B lineage cells in the absence of IL-7 (34).

CLPs developed in an IL-7-deficient context possess normal T/NK (natural killer) differentiation potential. However, their B differentiation potential is severely impaired. In limiting dilution assays, CLPs cultured in conditions favorable for B lymphopoiesis generated B lineage cells more than CLPs isolated from IL-7^−^ mice cultured in the same conditions. In contrast, enforced expression of EBF (a type of B lineage transcription factor) into CLPs from IL-7^−^ mice made it possible to restore their capacity to differentiate into B lineage cells (35). Notably, IL-7R signaling has been demonstrated to lead to the expression of EBF by activation of STAT5, a major signaling molecule downstream of the IL-7R signaling pathway. Therefore, IL-7 receptor signaling acts as an important component in forming the transcription factor network during B lymphopoiesis *via* upregulating EBF, allowing stage transition from the pre-pro-B to further maturational stages (36).

IL-7 is essential for murine B-cell development. However, unlike in mice, the development of human B cells appears to proceed typically in the absence of IL-7. A gene mutation located at the human γc locus may lead to a disease called X-linked severe combined immunodeficiency (X-SCID), characterized by a deficiency of T and NK cells in the presence of normal quantities of B cells (10). Indeed, IL-7 reacts with B-cell precursors to show higher survival and proliferation ability by mediating STAT5 (37). Although neonatal cord blood can produce B-cell progenitor cells in the absence of IL-7, IL-7 greatly increases the production of B cells in co-cultures containing human BM stroma and either adult BM HSCs or cord blood (38). Experiments also revealed the crucial effect of IL-7 on peripheral B-cell numbers. For example, a transient decrease of peripheral B cell numbers could be observed after IL-7 therapy, normalizing several weeks after the initiation of treatment. This further suggested that B-cell lymphopoiesis may be affected by the IL-7 levels of peripheral blood (39). The selective cytokine culture experiments conducted by Bruno et al. confirmed that the production of human B-line cells outside the fetal stage depends on the signal mediated by IL-7Rα, which could be provided by IL-7 or TSLP. The effectiveness of IL-7 on B lymphopoiesis *in vivo* was demonstrated by the decrease in human B cell progenitor cells after treatment with IL-7 neutralizing antibody in xenografts (40). In addition, the high expression level of IL-7 was reported to be responsible for the increased proportion of immature transitional B cells in patients infected with HIV-1 (39, 41, 42).

More recently, experiments by Yu et al. found that a PLCγ1/PLCγ2 double deficiency in mice resulted in the developmental arrest of early B cells and rendered B-cell progenitors irresponsive to IL-7. Inhibition of mammalian target of rapamycin (mTOR) activation induced by PLCγ/PKC impaired IL-7-mediated B-cell development. Briefly, IL-7 receptors regulated early B lymphopoiesis by activating the mTOR *via* PLCγ/DAG/PKC signaling (43). Despite its positive effect on B-cell production, IL-7 has been demonstrated to be an unfavorable prognostic factor affecting clinical outcomes in both mice and humans. For example, compared with healthy individuals, patients with Hodgkin’s lymphoma display higher serum levels of IL-7, and IL-7 mRNA-specific signals are detectable in tumor tissues (44). Lymphomas are also frequently observed in IL-7 transgenic mice (45). The role of IL-7 in the pathogenesis of types of lymphoma and leukemia is documented in several studies (46, 47).

### IL-7/IL-7R and B-Cell Survival

IL-7 promotes B-cell survival by modulating pro-apoptotic production (such as Bax, Bad, and Bim) and anti-apoptotic factors (such as Bcl-2, Bcl-xL, and Mcl-1). Studies have shown that different regions of the IL-7 receptors initiated the signal transduction pathways that regulate the Bcl-2 family, including the synthesis of Bcl-2, phosphorylation of Bad, and cytosolic retention of Bax (48). Short-term culture of immature thymocytes with IL-7 causes an increase in Bcl-2 expression and cell survival (49). Mcl-1 is another critical factor associated with the survival of B cells, and STAT5 regulates its expression directly (50). Defects in Mcl-1 expression increase apoptosis of B cells and arrest the development at the pro-B-cell stage. In thymocytes deficient in recombination activating gene 2, exposure to IL-7 stimulation leads to a significant increase in Mcl-1mRNA levels within 30 min (51). PI3K/Akt and JAK/STAT pathways also play an important role in mediating the survival responses of IL-7. PI3-K initiates Akt-dependent phosphorylation of Bad, which is conducive to maintaining Bad in the cytosol. To prevent apoptosis, this procedure requires the activation by IL-7R signaling (52). Bax is a significant pro-apoptotic factor in B-cell development, and mice lacking the signaling component JAK3 or IL-7R display greatly increased Bax levels (53, 54). More interestingly, although B cells are insensitive to IL-7 (due to the lack of expression of IL-7R on mature B cells), high concentrations of IL-7 promote B-cell survival and increase antibody production in the presence of T cells without using any other B-cell stimulatory signal. The mechanism is that IL-7 promotes B cell activation through stimulating expression of CD70 on CD4^+^ memory cells. IL-7 treatment also triggers resting peripheral T cells to secrete BAFF, thus promoting the survival of B cells (55).

## IL-7/IL-7R and NK Cells

Human NK cells comprise approximately 15% of all circulating lymphocytes. In humans, NK cells can be divided into two subsets: CD56^bright^ and CD56^dim^ subsets, based on their localization and the cell-surface density of CD56 (56). The two subsets have distinct functional properties. The CD56^bright^ NK population produces large amounts of diverse cytokines. In contrast, CD56^dim^ NK population possesses high cytotoxic activity. We already know that CD127 is expressed predominantly on CD56^bright^ NK cells (57). More importantly, CD127 acts as a molecular marker in the development of mouse NK cells derived from the thymus. CD127^+^ NK cells originating from the thymus repopulate in peripheral lymphoid organs, where IL-7 strictly regulates their homeostasis (58). Studies report that IL-7 has redundant functions for generating NK cell precursors and immature NK cells. It also plays a critical role in the normal homeostasis of mature NK cells in the spleen (59–61).

Vosshenrich et al. compared the generation of thymic NK cells in Rag2^-^IL7^+^ and Rag2^-^IL7^-^ mice. The phenotype and absolute number in the spleen and BM were not significantly different. However, mice lacking in IL-7 had rare thymic CD127^+^NK cells, indicating that IL-7 is critical for the homeostasis (58). The authors assessed the NK cell number that the thymus contributed to the peripheral NK cell pool and found that considerable CD127^+^ NK cells were exported to peripheral organs (58). However, in contrast to observations in mice, Michaud et al. assessed the IL-7Rα expression levels in mature NK cells isolated from human peripheral blood, and found that IL-7Rα^+^CD56bright NK cells were independent of thymic maturation because the NK cells extracted from athymic patients expressed IL-7Rα. Moreover, the team confirmed that IL-7 enhanced the survival of CD56^bright^NK cells by increasing the expression of Bcl-2 (57).

Several lines of evidence confirm that IL-7 is extremely important in disease control through regulating the biological functions and homeostasis of NK cells. For example, patients with multiple sclerosis (MS) have decreased NK cell numbers and impairment of NK cell functions. The levels of IL-7 and IL-7Rα in MS patients affect the functional responsiveness of NK cells. IL-7 induces an increase of IFN-γ production in CD56^bright^ NK cells and a pronounced enhancement of cytotoxicity in NK cells from patients with MS (62). In hepatitis C virus (HCV) mono-infection and HIV–HCV co-infection, IL-7 enhances NK-cell degranulation and promotes NK-cell cytolysis of target cells (63). Correspondingly, by using CD3^-^CD16^+^CD56^+^ cells from HIV-positive and -negative donors, Lum et al. showed that IL-7 could augment NK function by upregulating Fas ligand (64).

## IL-7/IL-7R and ILCs

Innate lymphoid cells (ILCs) are a recently discovered family of lymphoid cells important for eliminating external pathogens, tissue development and remodeling, and immune defense at multiple mucosal sites (65). ILCs are categorized into three broad classes: ILC1s, ILC2s, and ILC3s. A further subset of ILCs is LTi, namely, lymphoid tissue inducer cells. NK cells are similar to ILC1s but are not considered part of the ILC subset. IL-7 is involved in the development of all ILC subsets, as demonstrated by animal experiments. For instance, compared with wild-type (WT) mice, only a marginal reduction of ILC1s was observed in IL-7R^-^ mice or IL-15^−^ mice. In contrast, ILC1s in IL-7R^–^IL-15R^-^ mice were significantly reduced, indicating a synergistic effect from IL-7 in maintaining ILC1s (66).

One study suggested that IL-7 is critical for the survival and maintenance of ILC2s in the tissue (67). The dependency of transcription factors may shed light on the mechanism of IL-7’s mediation in developing ILCs. Id2^+^CHILPs (common helper innate lymphoid precursor cells) can generate ILC subsets (ILC1s, ILC2s, ILC3s, and LTi), and a transcription factor called NF1IL3 has been confirmed as indispensable for the generation of CHILPs and expression of Id2 (68). IL-7 promotes the expression of NFIL3 (68), and therefore, a deficiency in IL-7 impairs the development of all ILC subsets. In addition, IL-7 and its receptor drive the differentiation and generation of ILCs by initiating the expression of transcription factor Sall3 in CHILPs (69). Recent work by Yang et al. emphasized the importance of IL-7 for the development of ILC3s, and found that IL-7-dependent maintenance of ILC3s resulted in the normal entry of lymphocytes into lymph nodes (70).

## IL-7/IL-7R and Monocytes/Macrophages

Information about the effects of IL-7 on monocytes is sparse and the role of IL-7 in the development of CD14^+^monocytes is not yet clarified. Studies from the last century described the destruction of monocyte-derived macrophages (MDMs) infected with Mycobacterium avium mediated by IL-7 (71). The anti-tumor cytotoxic and antimicrobial effects of monocytes/macrophages treated with IL-7 have also been described (71, 72). In patients with autoimmune disorders, IL-7 combined with blood monocytes to maintain human CD4^+^ memory cells with mixed regulatory/helper functions (73). Recently, scientists have reported studies on the *in vitro* effects of IL-7. An increased HLA-DR expression of monocytes in the presence of IL-7 has been reported. Thus, increased antigen presentation may improve the monocyte effect mechanism *in vitro* (74). Li et al. demonstrated that administration of IL-7 *in vivo* significantly augmented the activity of lung-resident purified monocytes. Treatment with IL-7 resulted in elevated STAT5 phosphorylation, increased pro-inflammatory cytokine secretion, and promotion of cellular proliferation. Activation of CD4^+^T cells was induced by monocytes and further enhanced after treatment with IL-7 (75). IL-7 also induced the recruitment of monocytes to the endothelium and promoted the cytokine secretion of CD14^+^monocytes (9, 72, 76). However, the biological relevance of these findings remains elusive because of the relatively low IL-7R expression in monocytes and the indirect effects of other cells and cytokines.

## IL-7/IL-7R and Dendritic Cells

Dendritic cells (DCs) have long been recognized as important components of immune cells. To date, there is no unified view on the role of IL-7 in DCs. Katz and Takeuchi found that DCs were independent of IL-7, and IL-7R was not required to develop DCs (77). In their experiments, IL-7R^-^BM cells were transferred into sub-lethally irradiated WT mice. They found that IL-7Rα knockout (KO) cells reconstituted various DC subsets, and thymic, splenic, peripheral lymph nodes (pLN) and thymic-plasmacytoid DCs were reconstituted by IL-7RαKO and WT donor cells. However, their study was limited by the single experiment design and difficulties in determining the proportion of DCs from donors. In contrast to Katz and Takeuchi’s work, Vogt et al. used multiple *in vivo* models. Each model lacking in IL-7 demonstrated reduced DC numbers, strongly suggesting that precursors of both conventional DCs and plasmacytoid DCs depended on IL-7 (78). The addition of IL-7 to fetal thymus organ cultures (FTOC) led to the continuous generation of large numbers of functional DCs. Nevertheless, endogenous deficiency of IL-7 reduced DC numbers drastically (79). Saunders et al. noted that after a 4-day culture period with a mixture of several cytokines, including IL-7, the mice thymic precursors proliferated and differentiated to DCs instead of T-lineage cells (80). Marquez et al. considered that human intrathymic precursors can differentiate to T-lineage cells if cultured in the presence of IL-7, and can then simultaneously develop into both monocytes and DCs (81). To date, there is limited research on the precise mechanism of IL-7 in DC development. However, granulocyte-macrophage colony-stimulating factor (GM-CSF) was confirmed to regulate the development of cDCs (conventional dendritic cells) and pDCs (plasmacytoid dendritic cells) by employing STAT5 to inhibit the IRF8 and the transcriptional network in lin^-^Flt3^+^ progenitors (82). IL-7 is known to trigger the phosphorylation of STAT5 (83, 84), and thus, it seems likely that IL-7R could be an important signal upstream of STAT5 in DC precursors.

Moore et al. (85) set out to elucidate the mechanism of IL-7’s influence on DC biology. They examined the corticomedullary structure and DC populations in IL-7R^-^ thymus, showing that a loss of IL-7R^–^dependent cells led to an inverted ratio of medullary thymic epithelial cells (mTECs) to cortical thymic epithelial cells (cTECs). An impact on the accumulation of three thymic DC subsets was also noted. Their BM chimera experiments revealed that the deficiency in the DC compartment from IL-7R^-^ thymus is cell-extrinsic. Therefore, although there is no intrinsic need for IL-7 during the development of DCs derived from thymic tissue, IL-7 is extremely important for establishing microenvironments that allow the accumulation of thymic DCs.

## IL-7/IL-7R and Neutrophils

IL-7 receptors are also expressed in neutrophils, but their role in neutrophil biology has attracted less attention from scientists compared with other immune cells. In the last century, researchers found that intravenous injection of IL-7 into mice increased neutrophils (86, 87). Jiang et al. introduced the IL-7R gene into IL-7R^-^ BM progenitors to test the feasibility of IL-7R transgenic therapy. An unanticipated result was the almost logarithmic increase in neutrophils (88). In addition to affecting the number of neutrophils, IL-7 treatment has been reported to accelerate the recruitment of neutrophils by promoting T-cell IL-17 secretion (8). This is because IL-17 acts on mesothelial cells to trigger the secretion of CXCL1/KC and CXCL2/MIP-2 (89), which have been demonstrated as essential for the promotion of neutrophil recruitment and granulopoiesis (90, 91). However, IL-7 only accelerated neutrophil recruitment, and the acceleration of its activation or functionality was not observed in the study (8).

## IL-7/IL-7R and Eosinophils

There are few reports of responsiveness to IL-7 by eosinophils. Vellenga et al. reported CD127 expression on eosinophil progenitors in BM and showed that IL-7 promoted eosinophil colony formation from human BM cells (92). In addition, mRNA for CD127 and CD132 were found to be expressed in human blood eosinophils (93). Some primary studies have indirectly confirmed that IL-7 can increase the production of eosinophils. For example, eosinophil infiltration has been observed in mice colonic mucosa after being treated with overproduction of IL-7 in the colon (94). Similar results have been reported in murine tumors engineered to overexpress IL-7 (95, 96). Conversely, a lack of eosinophils was observed in mice treated with targeted IL-7 deletion (97). Western blotting analysis by Kelly et al. confirmed the existence of IL-7Rα in highly purified human blood eosinophils and revealed its novel property of upregulating the activation marker CD69. More importantly, it demonstrated that IL-7 promotes the survival of human eosinophils (98).

### Clinical Studies on IL-7 Therapy

Clinical studies with IL-7 consistently demonstrates effective results, especially for acute and chronic infectious diseases. For example, a case report of a patient with progressive multifocal leukoencephalopathy (PML) showed that IL-7 decreased circulating John Cunningham (JC) virus, rapidly increased lymphocytes, and contributed to disease resolution (99). Sepsis is a perennial problem, but many high-profile and cutting-edge therapies are ineffective for sepsis management. The first trial of immunoadjuvant therapy targeting defects in adaptive immunity in septic patients demonstrated that IL-7 restored lymphocytes in septic shock (100). Encouragingly, IL-7 therapy also proved effective for novel coronavirus disease (COVID-19), which represents the greatest medical challenge in decades. In a recent case series, 12 critically ill patients with COVID-19 and severe lymphopenia were treated with IL-7 therapy. The lymphocyte count of the IL-7 group was more than double that of the control group (101).

Highly active antiretroviral therapy (HAART) has been recognized as effective in the treatment of HIV infection. However, while HAART almost completely inhibits viral replication, it fails to restore immune function. Preliminary clinical trial results demonstrated that IL-7 therapy improved proliferation and survival of T cells in HAART-treated HIV^+^ individuals (102). This suggests that HAART may translate into more favorable clinical outcomes with the use of IL-7. Similarly, Sereti et al. confirmed that IL-7 administration drove T-cell cycle entry and expansion in HIV-1 infection (103). Moreover, administration of recombinant human interleukin-7 (rhIL-7) improved the gut mucosal abnormalities of chronic HIV infection and attenuated the systemic inflammatory and coagulation abnormalities linked to it (104).

In addition to IL-7’s application in infectious diseases, current understanding of cancer immunotherapy suggests that IL-7 therapy has great potential for cancer treatment. In the first clinical trial with humans, 16 patients with refractory cancer were treated with rhIL-7 every other day for a total of 14 days. Substantial dose−dependent increases in the numbers of circulating CD4^+^ and CD8^+^ T cells were observed in the trial, with increases peaking at 3 weeks after the IL-7 therapy and being sustained for at least 2 months (105). In lymphopenic metastatic breast cancers, rhIL-7 administration before chemotherapy significantly increased CD4^+^ and CD8^+^ T-cell counts, but there was no obvious increase in the expression level of inflammatory cytokine (20). Preclinical studies have validated the anti-tumor potency of IL-7 therapy. Intra-tumoral delivery of IL-7-transduced DCs resulted in increased production of interferon (IFN) and GM-CSF, thereby inducing superior antitumor responses (106). In addition to using IL-7 alone, efforts to combine IL-7 with other molecules have also occurred. A group of researchers demonstrated that therapy using an IL-7 complex, formed with an IL-7R-Fc, induced anti-tumor responses by increasing tumor infiltration of T cells through CXCR3 chemokine signaling (107).

## Conclusion and Unanswered Questions

The subject of IL-7 function and regulation is challenging and highlights the complexity of this cytokine. Several general conclusions can be drawn from the review. First, most types of immune cells are rigorously regulated by IL-7 throughout their lifespan. Second, although common effects exist, the ultimate influence of IL-7 regulation differs according to the cell type. Finally, the expression of IL-7 positively regulates the expression of pro-inflammatory cells and cytokines, indicating that the application of IL-7 is a promising therapeutic strategy for many diseases.

Several unanswered questions and challenges remain to be solved. For instance, the cells induced to secrete IL-7 during immune responses to specific diseases are not known, and we do not yet know the appropriate amount of IL-7 for different stages of diseases. When is the most suitable time to apply IL-7 to enhance immune reconstitution after infection with a specific pathogen? What is the hierarchy of transcription factor binding to IL-7 regulatory elements in different types of cells? However, many of these questions may be solved by further animal experiments and clinical trials, and combining traditional biochemical methods and high-throughput approaches to clarify molecular signal transduction pathways.

## Author Contributions

DC and Z-HT participated in the design and drafted the manuscript. T-XT, HD, and X-PY participated in critical discussions and revised the manuscript. Z-HT supervised the project. All authors contributed to the article and approved the submitted version.

## Funding

This work was supported in part by the National Natural Science Foundation of China 81873870 (Z-HT).

## Conflict of Interest

The authors declare that the research was conducted in the absence of any commercial or financial relationships that could be construed as a potential conflict of interest.

## Publisher’s Note

All claims expressed in this article are solely those of the authors and do not necessarily represent those of their affiliated organizations, or those of the publisher, the editors and the reviewers. Any product that may be evaluated in this article, or claim that may be made by its manufacturer, is not guaranteed or endorsed by the publisher.
